# Why Age Matters to Higher Education: Age-Friendly Tools and Techniques for Culture Change

**DOI:** 10.1093/geroni/igaa057.1804

**Published:** 2020-12-16

**Authors:** David Burdick, Karen Rose, Dana Bradley

## Abstract

Momentum is growing for the Age-Friendly University Network as proponents, primarily gerontology educators, have successfully encouraged university presidents to sign nonbinding pledged to become more age-friendly in programs and policies, endorsing 10 Age-Friendly University Principles. While this trend is inspiring, more is needed to fully achieve benefits for universities, students, communities, and older adults. Four presentations discuss innovative ways of deepening university commitment, weaving the principles into the fabric of the university. The first paper describes thematic content analysis from five focus groups with admissions and career services staff at Washington University in St. Louis and the recommendations that emerged for the provision of programs and services for post-traditional students. The second paper describes efforts to utilize community-impact internships and community partnerships to build support for Age-Friendly University initiatives at Central Connecticut State University, particularly in the context of the university’s recent Carnegie Foundation Engaged Campus designation. The third paper describes how Drexel University became Philadelphia’s first Age-Friendly University and current efforts in the Drexel College of Nursing and Heatlh Care Profession’s AgeWell Collaboratory to convene university-wide leadership for an AFU Steering Committee working on four mission-driven efforts to ensure AFU sustainability. The fourth paper describes steps taken by AFU proponents at Western Oregon State University to gain endorsement from university leadership and community, including mapping the 10 AFU Principles to the university’s strategic plan, faculty senate endorsement, and survey/interview results of older community members’ use of the university, which collectively have enhanced deeper and broader campus buy-in of AFU.

